# Characteristics and outcomes of atrial fibrillation detected before and after acute ischemic stroke

**DOI:** 10.1007/s00415-024-12671-z

**Published:** 2024-08-31

**Authors:** Lucio D’Anna, Michele Romoli, Kirsten Harvey, Eleni Korompoki, Roland Veltkamp

**Affiliations:** 1 Department of Stroke and Neuroscience, Charing Cross Hospital, Imperial College London Healthcare NHS Trust, Fulham Palace Road, London, W6 8RF UK; 2 Department of Brain Sciences, Imperial College London, London, UK; 3 Neurology and Stroke Unit, Department of Neuroscience, Bufalini Hospital, AUSL Romagna, Cesena, Italy; 4 Department of Clinical Therapeutics, National and Kapodistrian University of Athens, Athens, Greece; 5 Department of Neurology, Alfried-Krupp Krankenhaus, Essen, Germany; 6 Department of Neurology, University Hospital Heidelberg, Heidelberg, Germany

**Keywords:** Atrial fibrillation, KAF, AFDAS, Ischemic stroke

## Abstract

**Background:**

Atrial fibrillation (AF) can be known before the stroke (KAF) or be newly detected after stroke (AFDAS). It is unknown whether the outcome of stroke differs between KAF and AFDAS. We performed a propensity-matched analysis to investigate the outcome of patients with AFDAS and their counterparts with KAF.

**Methods:**

We analysed a consecutive series of patients enrolled into the EIDASAF study, a single centre, retrospective study of ischemic stroke patients with a diagnosis of AF before or after the event who had been admitted to the Hyperacute Stroke Unit of Imperial College Healthcare NHS Trust between 2010 and 2017.

**Results:**

Overall, our cohort included 959 patients with AF and acute ischemic stroke. After propensity score matching, 547 patients were matched (404 KAF group and 143 AFDAS group). The rates of in hospital death and of haemorrhagic transformation were significantly higher in KAF patients compared to AFDAS patients. Logistic regression analysis did not reveal a statistically significant influence of AF subtypes on the outcome of death. However, in logistic regression analysis KAF was associated with increased probability of haemorrhagic transformation (OR 9.64; CI 1.29–71.68, *p* = 0.022) after the index event.

**Conclusion:**

KAF is associated with an increased risk of haemorrhagic transformation but not of death when compared to AFDAS.

**Supplementary Information:**

The online version contains supplementary material available at 10.1007/s00415-024-12671-z.

## Introduction

Atrial fibrillation (AF) is associated with a fivefold increase in stroke risk. The prevalence of AF in ischemic stroke patients varies from 11 to 33% depending on the study design and on the methods used to detect AF [1–4]. AF can be newly detected in close temporal proximity after the index stroke (AFDAS) or can be known before the stroke (known or KAF) [5]. There is an ongoing debate whether KAF and AFDAS should be considered as separate entities with different patient characteristics and prognostic implications [6]. In patients with AFDAS, AF may have existed before the stroke but remained undetected. Alternatively, it may have arisen from the interplay of cardiogenic and neurogenic forces resulting from the brain damage [6, 7]. In previous observational studies, patients with AFDAS had a lower prevalence of coronary artery disease, congestive heart failure, prior myocardial infarction, cerebrovascular events, and a lower degree of cardiac abnormalities than those with KAF [8]. A recent meta-analysis of 21 studies comprising 22,566 patients revealed that the risk of recurrent stroke was 26% lower in AFDAS than in KAF while death rates did not differ between AFDAS and KAF [9]. However, the authors acknowledged that the rate of stroke recurrence and death of the patients were estimated without considering the differences in terms of vascular risk factors and cardiovascular comorbidities between patients with AFDAS and KAF and that adjusting for the relevant variables may have yielded different results [9]. Therefore, considerable uncertainty prevails regarding the implications of AFDAS vs. KAF on outcome of acute ischemic stroke.

In the present study, we performed a propensity-matched analysis to investigate the different impact of AFDAS and KAF on the risk of death, recurrent cerebrovascular events, hemorrhagic transformation, and rate of symptomatic intracranial haemorrhage (sICH) after acute ischemic stroke.

## Methods

This is an investigator-initiated, observational, retrospective single-centre study, that included stroke patients that were treated at the Hyperacute Stroke Unit (HASU) of the Imperial College Healthcare NHS Trust (ICHT) between 2010 and 2017. Data included in the present study were derived from the database of the EIDASAF study as previously published [10–12]. The EIDASAF study was approved by the Health Research Authority for collection of data within the NHS. The research did not require review by the UK Health Departments Research Ethics Service as it fell into the category of ‘Research limited to use of previously collected, non-identifiable information.’ Informed consent was not a legal requirement as the research was carried out using data collected as part of routine care and any researchers outside of the direct care team only had access to anonymised data. The EIDASAF database included patients with recent (< 4 weeks) ischemic stroke and AF that was either detected shortly after (AFDAS) or was already known before the index stroke (KAF).

### Definition of study variables

The following data were collected: age, sex, smoking status, history of alcohol abuse, history of hypertension (blood pressure > 140/90 mm Hg at least twice before acute stroke or already under treatment with antihypertensive drugs), history of uncontrolled hypertension (use of antihypertensive medication but ≥ 140/90 mmHg), history of diabetes mellitus (fasting glucose level > 126 mg/dL pre-prandial on two examinations, glucose level > 200 mg/dL postprandial, or HbA1c > 6.5% or under antidiabetic treatment), hyperlipidemia (total cholesterol > 200 mg/dL or triglyceride > 140 mg/dL or already on lipid-lowering therapy), history of symptomatic ischemic heart disease (myocardial infarction, history of angina, or previous diagnosis of multiple lesions on thallium heart isotope scan or evidence of coronary disease on coronary angiography), history of congestive heart failure, history of symptomatic peripheral arterial disease (intermittent claudication of presumed atherosclerotic origin; or ankle/arm systolic blood pressure ratio < 0.85 in either leg at rest, or history of intermittent claudication with previous leg amputation, reconstructive surgery, or angioplasty), previous stroke, transient ischemic attack (TIA), previous intracranial hemorrhage (ICH), history of dementia and pre-stroke modified Rankin Scale (mRS). Data on the use of any antiplatelets and anticoagulants, before admission, at baseline, and during the admission period, were recorded. The CHA_2_DS_2_-VASc score was calculated after the index event. Treatment with intravenous thrombolysis and mechanical thrombectomy for the index stroke was documented. Direct oral anticoagulant (DOAC) therapy was defined as one of the following drugs and dosages: apixaban 2.5 mg or 5 mg twice daily; dabigatran 110 mg or 150 mg twice daily; edoxaban 30 mg or 60 mg once daily; or rivaroxaban 15 mg or 20 mg once daily. Vitamin K antagonist (VKA) was defined as treatment with acenocoumarol/warfarin. The choice of the anticoagulant treatment (pre and post the index event) was decided by the treating physician as part of routine clinical care. We collected data on systolic and diastolic blood pressure on admission.

To identify patients with AFDAS, we used multiple overlapping sources. In first instance, we took the history from the patient and whether this was not possible the history was taken from the next of kin or any family member. Secondly, we reviewed all previous hospital discharge records, and we contacted all the general practitioners (GPs) by telephone and e-mail to invite them to give information about the medical history of the patients included. Finally, we checked the NHS summary care records to search for any record of previous atrial fibrillation. AFDAS was diagnosed with a baseline 12-lead ECG or with bedside continuous electrocardiogram monitoring using standard Philips monitors (software version B.02.12, Amsterdam, Netherlands). This monitoring system includes different rhythm alarms (flat ECG, pre-set upper and lower heart rate threshold, nonsustained ventricular tachycardia and ventricular fibrillation). Stroke physicians and nurses on the HASU are trained to recognize and document any episodes suspicious for atrial fibrillation on the monitor. When atrial fibrillation was suspected on monitor recordings, a 12-lead ECG was performed and reviewed by a trained stroke physician.

### Classification of competing stroke aetiologies

Two trained investigators reviewed the clinical history, neurological examination, diagnostic investigations, and brain imaging studies of all enrolled patients and assigned infarct subtype classifications following the Trial of ORG 10172 in Acute Stroke Treatment (TOAST) criteria [13, 14]. As all stroke patients included in the current study had AF, we documented any additional competing stroke aetiology which was classified using the following categories: large-artery atherosclerosis (including large-artery thrombosis and artery-to-artery embolism); small-vessel occlusion; and stroke of other determined cause. Patients were classified as having a competing stroke aetiology due to large-artery atherosclerosis if their clinical and brain imaging showed either a hemodynamically relevant (> 50%) stenosis or an occlusion of an extracranial or intracranial artery, presumably due to atherosclerosis. Small-vessel occlusion was considered as competing aetiology when they presented with a lacunar syndrome or had a brainstem or deep hemispheric infarct with a diameter of less than 1.5 cm demonstrated. The category ‘stroke of other determined cause’ included patients with rare causes of stroke, such as nonatherosclerotic vasculopathies, hypercoagulable states, or hematologic disorders. The classification into one or several of these subtypes was based on clinical features and results of diagnostic tests including neuroimaging and vascular imaging studies, cardiac tests, electrocardiogram (ECG), echocardiography and assessment of prothrombotic syndromes. When we did not find any other possible competing aetiology related to the index event, we considered cardioembolism as the only probable cause of the event. When essential studies such as brain imaging, vessel evaluation, echocardiography, and ECG were not performed, we classified these patients as non-determined aetiology for incomplete evaluations.

### Neuroimaging analysis

All cranial images performed on admission, either cranial computed tomography (CT) and magnetic resonance imaging (MRI), were analysed centrally according to a prespecified protocol, by experienced stroke researchers, to describe the size and the location of the infarct and to attribute the most likely causative mechanisms. This lesion was delineated manually using MRICroN software (version 12/2009; C. Rordens) [15] in native space to obtain the volume. Mature lesions and leukoaraiosis were excluded. The acute ischemic lesions were classified by their vascular territory: anterior cerebral artery (ACA), middle cerebral artery (MCA), posterior cerebral artery (PCA), lacunar, brainstem and cerebellar infarcts. MCA infarcts were divided into superficial (involving the cortex and the underlying white matter), deep (involving the basal ganglia, the internal capsule and the deep white matter) and combined. We collected data on the subcortical white matter hyperintensity (i.e. leukoaraiosis) rated according to the Fazekas scale. The Fazekas classification systems is a scale ranging from 0 (no WMD) to 3 (high WMD) [16].

### Study outcomes

The study outcome variables were death, recurrent cerebrovascular events, hemorrhagic transformation and rate of sICH during the inpatient admission. The study outcomes were identified by a careful search of each medical record including the discharge diagnosis by trained stroke physicians. Hemorrhagic transformation was defined on follow-up CT at 24 h as either small petechiae along the margins of the infarct (HI-1) or as more confluent petechiae within the infarcted area but without space-occupying effect (HI-2); as parenchymal hematoma (PH) defined as hematoma in < 30% of the infarcted area with some slight space-occupying effect (PH-1) or as dense hematoma in ≥ 30% of the infarcted area with substantial space-occupying effect or as any hemorrhagic lesion outside the infarcted area (PH-2). In the case of more than one hemorrhagic lesion on brain scan, the worst possible category was assumed. Hemorrhagic transformation was considered symptomatic (sICH) if it was not seen on the admission brain scan and there was, subsequently, a suspicion of hemorrhage or a decline in neurological status (i.e. an increase of more than 4 points in the NIHSS) [17, 18].

### Statistical analysis

Categorical variables are presented as count and percentage, continuous variables as mean and standard deviation or median and interquartile range according to normal distribution. A propensity-score matching (PSM) algorithm was implemented to mitigate potential differences across AFDAS and KAF groups regarding stroke severity, cardiovascular risk factors and type of treatment. The propensity score of the group variable (AFDAS vs KAF) was calculated for each patient, and a 1:4 nearest neighbour matching no-replacing algorithm was used to match patients within 0.2 × Standard Deviation (SD) of the logit of the propensity score according to their group status, with matching including variables diverging across groups. To determine whether the propensity score approach achieved balance in all potential confounders, we compared all baseline characteristics between the two groups before and after matching. Statistical comparisons were performed between patient groups using the *χ*2 test, Fisher exact test, Student t-test, and Mann–Whitney *U* as indicated for dichotomous or continuous variables. Backward stepwise binary logistic regression was implemented to weigh the impact of AFDAS on primary outcome. Logistic regression was modelled for the primary outcome depending on factors emerging from univariate analysis, with matched variables and AF group implemented a priori.

## Results

The EIDASAF study included 959 consecutive patients with AF and acute ischemic stroke. Patients were followed up in-hospital with an average time of 16.1 days. Among the included patients, 763 (79.6%) patients had KAF and 196 (20.4%) patients had AFDAS. Table 1 reports the baseline characteristics for the overall population and patients AFDAS and KAF before and after the PSM.

**Table 1:** Baseline characteristics in KAF vs AFDAS

		Before PSM			After PSM		
	Overall population (*n* = 959)	KAF (*n* = 763)	AFDAS (*n* = 196)	*p* value	KAF (*n* = 404)	AFDAS (*n* = 143)	*p* value
Age, years [mean, (sd)]	80 (6.5)	81 (6)	77 (7.5)	** < 0.001**	78.3 (9.9)	76.4 (10)	0.056
Female sex [*n*, (%)]	476 (49.6)	378 (49.5)	98 (50)	0.973	205 (50.7%)	73 (51.0%)	1
Smoker or ex-smoker [*n*, (%)]	243 (25.3)	178 (23.3)	65 (33.2)	**0.006**	99 (24.5%)	44 (30.8%)	0.381
Alcohol abuse [*n*, (%)]	97 (10.11)	71 (9.3)	26 (13.3)	0.131	48 (11.9%)	20 (13.9%)	0.611
Hypertension [*n*, (%)]	719 (74.9)	588 (77)	131 (66.9)	**0.004**	292 (72.3%)	102 (71.3%)	0.233
Diabetes [*n*, (%)]	218 (22.7)	178 (23.3)	40 (20.4)	0.438	89 (22.0%)	32 (22.4%)	1
Hypercholesterolemia [*n*, (%)]	478 (49.8)	395 (51.8)	83 (42.3)	**0.023**	179 (44.3%)	62 (43.4%)	0.921
Ischemic heart disease [*n*, (%)]	267 (27.8)	228 (29.9)	39 (19.9)	**0.007**	93 (23.0%)	27 (18.9%)	0.362
Heart failure [*n*, (%)]	116 (12.1)	107 (14)	9 (4.6)	** < 0.001**	29 (7.2)	7 (4.9)	0.453
Peripheral vascular disease [*n*, (%)]	77 (8)	60 (7.9)	17 (8.7)	0.822	21 (5.2)	12 (8.4)	0.240
Previous IS [*n*, (%)]	229 (23.9)	206 (27)	23 (11.7)	** < 0.001**	73 (18.1%)	19 (13.3%)	0.236
Previous TIA [*n*, (%)]	126 (13.1)	109 (14.3)	17 (8.7)	**0.050**	60 (14.9)	12 (8.4)	0.069
Dementia [*n*, (%)]	115 (12)	102 (13.4)	13 (6.6)	**0.013**	33 (8.2)	7 (4.9)	0.269
CHA_2_DS_2_-VASC score [median (IQR)]	6 (5–6)	6 (5–6)	5 (4–6)	** < 0.001**	5 (5–6)	5 (4–6)	0.225
NIHSS on admission [median (IQR)]	6 (2–13)	6 (3–14)	4 (2–8.5)	** < 0.001**	5 (2–11)	4 (2–8.5)	0.089
Intravenous thrombolysis [*n*, (%)]	147 (15.3)	104 (13.6)	43 (21.9)	**0.005**	68 (16.8%)	35 (24.5%)	0.060
Mechanical thrombectomy [*n*, (%)]	25 (2.6)	18 (2.4)	7 (3.6)	0.484	16 (3.9%)	11 (7.7%)	0.854
Antiplatelets [*n*, (%)]	326 (34)	257 (33.7)	69 (35.2)	0.751	129 (31.9%)	57 (39.9%)	0.106
Oral anticoagulant [*n*, (%)]	294 (30.7)	283 (37.1)	11 (5.6)	** < 0.001**	145 (35.4%)	11 (7.6%)	** < 0.001**
*VKA *[*n*, (%)]	220 (23)	209 (27.4)	11 (5.6)		112 (27.7)	9 (6.3)	
*DOAC *[*n*, (%)]	74 (7.7)	73 (9.6)	1 (0.5)		33 (8.2)	2 (1.4)	
Systolic blood pressure on admission, [median (IQR)]	142 (123–160)	145 (129–165)	143 (131–164)	0.699	149 (132–167)	149 (131–166)	0.547
Diastolic blood pressure on admission, [median (IQR)]	77 (70–90)	79 (71–91)	82 (72–94)	0.320	80 (70–92)	82 (72–956)	0.064
In hospital length of stay, days [median (IQR)]	16.1 (4–28)	16.4 (3–26)	15.8 (4–27)	0.560	16.2 (4–27)	16 (3–28)	0.583

*AFDAS* atrial fibrillation newly detected in close temporal proximity to the index stroke, *KAF* atrial fibrillation known before the stroke, *PSM* propensity-matched analysis, *IS* ischemic stroke, *TIA* transient ischemic attack, *ICH*: intracranial haemorrhage, *mRS* modified Rankin Scale, *NIHSS* national institutes of health stroke scale

**Fig. 1:**
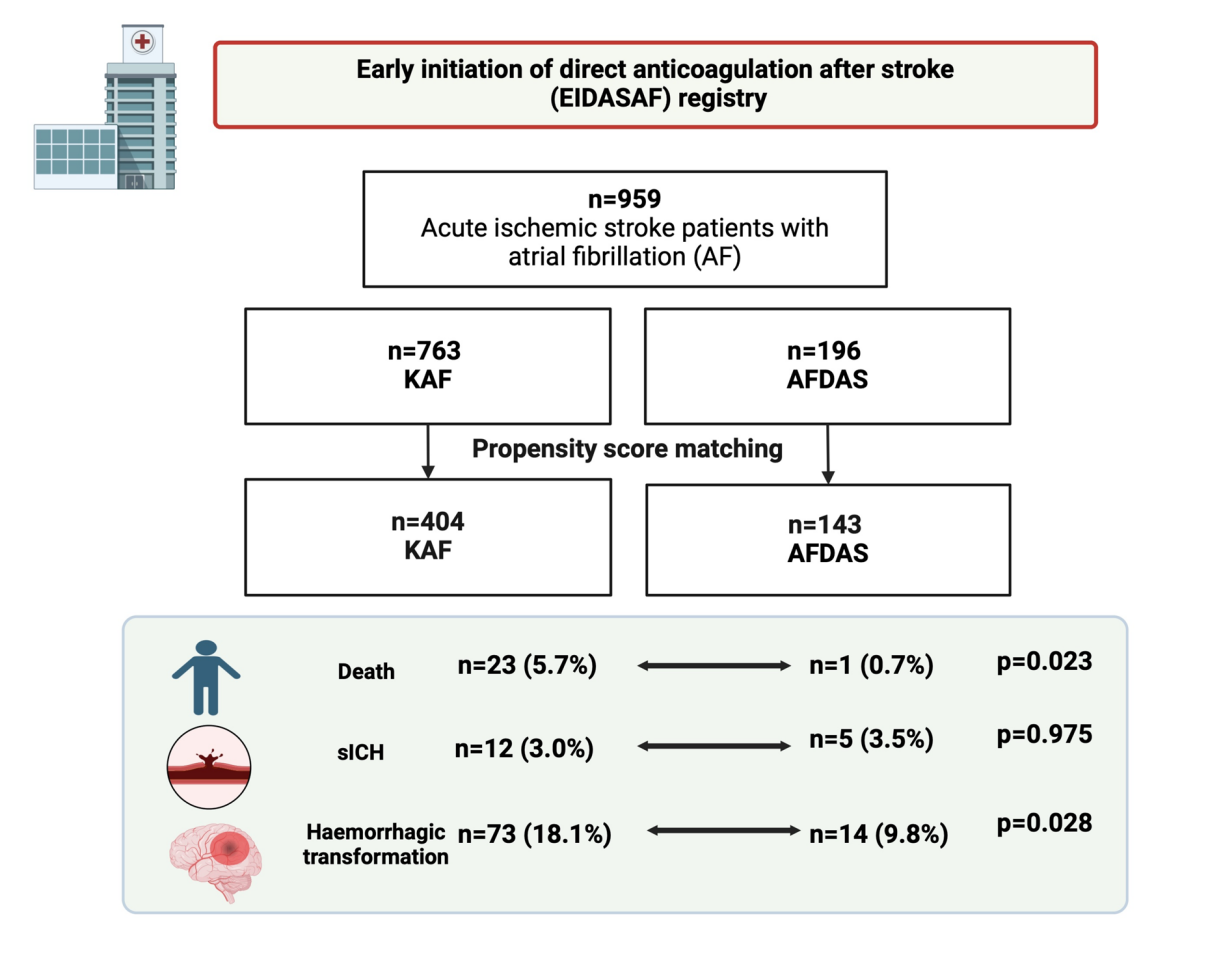
Study algorithm

After PSM, 547 patients were matched (Fig. 1, Supplemental Table 1). There were 404 patients with KAF (73.9%) and 143 with AFDAS (26.1%). After PSM, between the two groups differed significantly only in terms of use of oral anticoagulant therapy prior to the index event (*p* < 0.001). Of note, 11 out of 143 AFDAS patients (7.6%) were on treatment with oral anticoagulant because of previous deep venous thrombosis.

Table 2 presents data on the competing stroke aetiologies in the two groups of patients. Among the 404 KAF patients, 200 (49.5%) had another competing cause of their stroke in addition to AF-related cardioembolism compared to 42 (29.4%) of AFDAS patients (*p* < 0.001). AFDAS patients more often did not have any other competing aetiology compared to KAF patients (*p* < 0.001). In contrast, infarct volume, Fazekas score and location of the index infarct did not differ between the two groups (Table 3).

**Table 2:** Competing stroke aetiologies in KAF vs AFDAS

	KAF (*n* = 404)	AFDAS (*n* = 143)	*p* value
Any other competing etiology [*n*, (%)]	200 (49.5)	42 (29.4)	** < 0.001**
LAA [*n*, (%)]	100 (24.8)	17 (11.9)	
Lacunar [*n*, (%)]	77 (19.1)	23 (16.1)	
Other etiologies [*n*, (%)]	23 (5.7)	2 (1.4)	
No other competing aetiology demonstrated—probable only cardioembolic aetiology *n*, (%)]	103 (25.5)	63 (44.1)	** < 0.001**
No determined for incomplete investigations *n*, (%)]	101 (25.4)	38 (26.4)	0.795

*AFDAS* atrial fibrillation newly detected in close temporal proximity to the index stroke, *KAF* atrial fibrillation known before the stroke, *LAA* large artery atherosclerosis

**Table 3:** Location and characteristics of ischemic stroke in KAF vs AFDAS patients

	KAF (*n* = 404)	AFDAS (*n* = 143)	*p* value
Infarct volume [median (IQR)]	5.54 (0.87–24.8)	4.9 (0.87–22.9)	0.940
Fazekas score [*n*, (%)]			0.641
0	277 (68.6%)	92 (64.3%)	
1	52 (12.9%)	18 (12.6%)	
2	51 (12.6%)	24 (16.8%)	
3	23 (5.9%)	9 (6.3%)	
ACA [*n*, (%)]	12 (3.0)	5 (3.5)	0.999
Large MCA [*n*, (%)]	134 (33.2)	54 (37.8)	0.533
Superficial MCA [*n*, (%)]	101 (25.0)	40 (28.0)	0.688
Deep MCA [*n*, (%)]	103 (25.5)	46 (32.2)	0.081
Combined MCA [*n*, (%)]	74 (18.3)	33 (23.1)	0.230
PCA [*n*, (%)]	43 (10.15)	13 (9.1)	0.651
Brainstem [*n*, (%)]	5 (1.2)	–	–
Cerebellum [*n*, (%)]	18 (4.5)	4 (2.8)	0.504

*AFDAS* atrial fibrillation newly detected in close temporal proximity to the index stroke, *KAF* atrial fibrillation known before the stroke, *ACA* anterior cerebral artery, *MCA* middle cerebral artery, *PCA* posterior cerebral artery

After the index event, anticoagulation was (re)-started or recommended to be started in 76.9% (*n* = 110) patients with AFDAS compared to 58.9% (*n* = 237) KAF patients during the hospital admission (*p* = 0.002) (Table 4). At discharge, we observed a statistically significant distribution of the choice of the anticoagulant for secondary prevention in the two groups of patients (*p* < 0.001). Moreover, there was a statistically significant difference between the median time of (re)-starting the anticoagulant therapy after the index event between the two groups of patients (*p* < 0.001).

**Table 4:** Anticoagulant management after index event in KAF vs AFDAS patients

	KAF (*n* = 404)	AFDAS (*n* = 143)	*p* value
Time to (re)-initiation of oral anticoagulant, days, [median (IQR)]	5 (2–11)	3 (2–9)	** < 0.001**
(re)-initiation of oral anticoagulant [*n*, (%)]	237 (58.9%)	110 (76.9%)	**0.002**
Choice oral anticoagulant ((re)-initiation)			** < 0.001**
VKA [*n*, (%)]	97 (24.0)	49 (34.3)	
DOAC [*n*, (%)]	136 (33.7)	57 (39.9)	
Heparin [*n*, (%)]	4 (0.9)	4 (2.8)	

*AFDAS* atrial fibrillation newly detected in close temporal proximity to the index stroke, *KAF* atrial fibrillation known before the stroke, *DOAC* direct oral anticoagulant, *VKA* vitamin K antagonist

The rate of death was significantly higher in KAF patients compared to AFDAS patients (*p* = 0.023) (Table 5). Table 6 shows the logistic regression analysis to identify the independent predictors of death after the index event. The univariate analysis showed that KAF (OR 8.57, 95% CI 1.15–65.05, *p* = 0.036) and (re)-initiation of oral anticoagulant after stroke (OR 0.08, 95% CI 0.02–0.25, *p* < 0.001) were significantly correlated with outcome of death after the index event. Use of oral anticoagulant pre index event, presence of any other competing etiology, no other competing etiology, time to (re)-initiation of oral anticoagulant and choice of anticoagulant were not correlated with outcome death after the index event. After multivariate analysis, the subtypes of AF were not statistically significantly associated with the risk of death.

**Table 5:** Study outcomes in KAF vs AFDAS patients

	KAF (*n* = 404)	AFDAS (*n* = 143)	*p* value
Death in hospital [*n*, (%)]	23 (5.7)	1 (0.7)	**0.023**
Recurrent TIA/Stroke [*n*, (%)]	2 (0.5)	–	–
Haemorrhagic transformation [*n*, (%)]	73 (18.1)	14 (9.8)	**0.028**
sICH [*n*, (%)]	12 (3.0)	5 (3.5)	0.975

*AFDAS* Atrial fibrillation newly detected in close temporal proximity to the index stroke, *KAF* atrial fibrillation known before the stroke, *sICH* symptomatic intracranial haemorrhage

**Table 6:** Logistic regression analysis for outcome of death

	Univariate analysis			Multivariate analysis		
	OR (95% CI)	*z*	*p*	OR (95% CI)	*z*	*p*
KAF	8.57 (1.15–65.05)	8.11	0.036	6.19 (0.78–48.78)	5.66	0.083
Use of oral anticoagulant pre index event	0.65 (0.24–1.77)	0.43	0.397	–	–	–
Presence of any other competing etiology	0.94 (0.38–2.31)	0.88	0.872	–	–	–
No other competing etiology demonstrated- probable only cardioembolic etiology	0.94 (0.38–2.31)	0.88	0.897	–	–	–
(re)-initiation of oral anticoagulant	0.08 (0.02–0.25)	0.01	< 0.001	0.01 (0.01–0.01)	0.99	0.996
Time to (re)-initiation of oral anticoagulant, days	0.91 (0.09–1.99)	1.64	0.099	–	–	–
Choice oral anticoagulant ((re)-initiation)						
VKA	0.06 (0.01–1.43)	0.07	0.106	–	–	–
DOAC	0.08 (0.2–1.38)	0.04	0.100	–	–	–
Heparin	0.01 (0.01–1.01)	0.98	0.991	–	–	–

*KAF* atrial fibrillation known before the stroke

The two groups did not significantly differ in terms of rate of sICH (*p* = 0.975) but a larger proportion of KAF patients had any haemorrhagic transformation (*p* = 0.028) (Table 5). Table 7 shows the logistic regression analysis to identify the independent predictors of haemorrhagic transformation after the index event. In logistic regression analysis, KAF was associated with increased probability of haemorrhagic transformation (OR 9.64, 95% CI 1.29–71.68, *p* = 0.022) after the index event. Use of oral anticoagulant pre index event, presence of any other competing etiology, no other competing etiology, (re)-initiation of oral anticoagulant,

**Table 7:** Logistic regression analysis for outcome of haemorrhagic transformation

	Univariate analysis		
	OR (95% CI)	*z*	*p*
KAF	9.64 (1.29–71.68)	9.14	**0.022**
Use of oral anticoagulant pre index event	0.69 (0.27–1.75)	4.61	0.758
Presence of any other competing etiology	1.35 (0.63–2.93)	2.14	0.409
No other competing etiology demonstrated- probable only cardioembolic etiology	0.83 (0.34–1.99)	6.53	0.387
(re)-initiation of oral anticoagulant	0.87 (0.62–1.21)	0.44	0.410
Time to (re)-initiation of oral anticoagulant, days	0.86 (0.43–2.16)	1.30	0.191
Choice oral anticoagulant ((re)-initiation)			
VKA	0.82 (0.27–1.97)	0.13	0.141
DOAC	0.63 (0.36–1.11)	0.21	0.116
Heparin	0.49 (0.06–4.16)	0.52	0.515

*KAF* atrial fibrillation known before the stroke

time to (re)-initiation of oral anticoagulant and choice of anticoagulant were not correlated with outcome of haemorrhagic transformation after the index event.

## Discussion

Our observational study found a similar early mortality in patients with KAF and AFDAS. There is a substantial heterogeneity and inconsistencies in the reported studies evaluating AF subtypes and post-stroke outcomes. Previous observational studies investigating outcome profiles of AFDAS and KAF in patients with acute ischemic stroke showed no differences in mortality between AFDAS and KAF despite a lower burden of vascular disease in the former group [9]. It was hypothesized that the risk of death in the two groups of patients is mainly driven by the severity of the stroke [9]. However, to date, only one other study by Watanabe et al. [19] used a propensity-matched analysis to adjust for possible confounding factors to investigate the outcomes of acute ischemic stroke patients with AFDAS compared to their counterparts with KAF. In this study, 64 patients in the AFDAS and KAF groups were matched [19]. There was no difference in the in-hospital outcome (mRS score at discharge) between the two groups and AFDAS was not a predictor of severity of mRS score at discharge. Unfortunately, in contrast to our study, the authors did not provide the rates of death between patients with AFDAS and KAF. In our analysis, the rate of in-hospital death was significantly higher in KAF patients compared to AFDAS patients. However, after the multivariable logistic analysis, whether AF was known before or diagnosed after stroke was not an independently associated with death.

In our propensity-matched analysis, the rate of hemorrhagic transformation was significantly higher in KAF patients compared to AFDAS patients, and KAF was an independent predictor of hemorrhagic transformation in patients with acute ischemic stroke. Only few previous studies have reported the incidence of hemorrhagic transformation in patients with AFDAS and KAF although the authors did not perform a propensity-matched analysis between the two groups. In the study of Hsieh et al. [20], the rates of hemorrhagic transformation were comparable between patients with AFDAS and KAF and in their multivariable analysis KAF was not independently associated with the composite outcome of ischemic stroke, hemorrhagic transformation, or death within 1 year after the index date. In contrast to our study, the authors did not explore singularly the outcomes one by one. Although we cannot determine the mechanism of higher rate of hemorrhagic transformation in KAF patients, they were more often taking an oral anticoagulant before the stroke which may have influenced the burden, location and clot composition and tendency for hemorrhagic transformation [21–25]. However, in our multivariable analysis, anticoagulation prior to the index event failed to be a predictor of hemorrhagic transformation after stroke. However, we acknowledge that in our study, we did not have access to detailed data on patient adherence to oral anticoagulant therapy, nor did we have information on the adequacy of treatment, such as International Normalized Ratio (INR) levels in patients on warfarin or drug concentrations in those on DOACs. This limitation is significant because poor adherence or suboptimal dosing could potentially lead to either under-anticoagulation, increasing the risk of ischemic events, or over-anticoagulation, raising the risk of ICH. Without data on INR values or DOAC levels, we cannot exclude the possibility that higher ICH rates in our cohort were associated with elevated anticoagulant levels, which could have contributed to the observed outcomes. Hemorrhagic transformation of the infarcts occurs in 5.9–50.8% of patients with acute ischemic stroke [26, 27]. While the majority of haemorrhagic transformations are asymptomatic and believed to be innocuous in some studies, haemorrhagic transformation was associated with adverse clinical outcomes such as mortality and disability in other studies [25, 28, 29]. Notably, the presence of haemorrhagic transformation is associated with a delay in the initiation of oral anticoagulation therapy after stroke, in patients with AF, which may increase the risk of early stroke recurrence [11, 29]. In our analysis, after the index event, anticoagulation was (re)-started in 76.9% patients with AFDAS compared to 58.9% KAF patients (*p* = 0.002). Moreover, there was a statistically significant difference between the median time of (re)-starting the anticoagulant therapy after the index event between the two groups of patients with a significantly longer interval in patients with KAF (*p* < 0.001). A better understanding of the factors underlying hemorrhagic transformation in patients with KAF may aid the early identification of patients at high risk of hemorrhagic transformation leading to a safer initiation of anticoagulation therapy in selected patients.

We acknowledge that the higher rates of mortality and sICH observed in patients with KAF may partly reflect the fact that this cohort tends to be older and generally presents with a greater burden of comorbidities, as highlighted in Table 1. Despite the use of PSM to balance observed covariates between the KAF and AFDAS groups, it is possible that residual confounding remains, particularly with respect to unmeasured or inadequately measured risk factors.

The non-significant statistical differences in certain variables post-matching suggest that the KAF group may still represent a population with a higher baseline risk profile, potentially contributing to the observed outcomes. For instance, factors such as frailty, polypharmacy, and the presence of multiple comorbid conditions, which are more prevalent in older populations, could exacerbate the risk of adverse outcomes following an ischemic stroke. This potential confounding effect underscores the need to interpret our findings with caution. It is essential to consider that the higher mortality and sICH rates in the KAF group might not be solely attributable to the type of atrial fibrillation but also to the overall health status of the patients. Future studies could benefit from incorporating more comprehensive adjustments for these factors or from focusing on subgroups of patients with more homogeneous health profiles to better isolate the effects of KAF versus AFDAS.

Another key finding of our analysis is that 145 (35.4%) out of 404 patients with KAF had an ischemic stroke while under anticoagulant treatment. Moreover, patients with KAF share most imaging characteristics of the patients with AFDAS. A variety of reasons may underlie the failure of anticoagulant treatment to prevent strokes in AF patients taking also into consideration that oral anticoagulant treatment cannot be considered fully effective in terms of stroke prevention. Of note, almost 49.5% of our KAF patients had another competing cause of their stroke in addition to AF compared to only 29.4% of AFDAS patients. We found that large artery disease or small vessel disease were the predominant competing causes of stroke in patients with KAF. Our results are in line with previous studies that documented a non-cardioembolic aetiology underlying ischemic stroke in patients with KAF such as large artery disease or small vessel disease [30, 31]. Our findings suggest that large artery disease or small vessel disease may represent the cause of ischemic stroke in a relevant proportion of patients with KAF under anticoagulant treatment. However, non-adherence to medication, under-dosage of anticoagulants, or their temporary discontinuation frequently can be associated with AF-related stroke despite anticoagulant treatment [10, 30]. Interestingly, a recent study by Lyrer et al. has showed that pre-existing anticoagulation rather than KAF was independently associated with a higher hazard for stroke recurrence [32]. Therefore, the authors recommended that future research should focus on the cause of stroke despite anticoagulation to develop improved preventative treatments. Hence, it is of paramount importance to investigate the individual circumstances of a stroke occurring on anticoagulants and to address the multiplicity of underlying factors in subsequent prevention.

Our study has several strengths including the use of propensity-matched analysis to adjust for possible confounding factors and a relatively large cohort of patients. Limitations encompass the retrospective data collection and the lack of longer term follow-up. We recognize that our study’s retrospective design introduces inherent limitations, particularly regarding selection bias and unmeasured confounding factors. Although PSM was employed to reduce these biases by balancing observed covariates between the AFDAS and KAF groups, it does not account for unobserved or unmeasured confounders. This limitation may impact the causal inferences drawn from our findings. However, in several circumstances in which randomized trials are not available, observational studies are considered a useful tool to understand the effects of a treatment or of different clinical services [33]. In addition, if rigorously designed, this type of analysis could help to estimate the effects of interventions, particularly in everyday clinical practice. However, despite our efforts, we cannot exclude that our results could have been influenced by an incomplete adjustment for patient characteristics in selecting the model for the multivariable analysis. Moreover, we do not have data on patients’ compliance to anticoagulation. Although our stroke service covers a multi-ethnic metropolitan area, the single-centre design may limit the generalizability of our findings. Finally, 25.4% of our patients had an incomplete diagnostic work up.

In conclusion, KAF is associated with an increased risk of hemorrhagic transformation but not of death when compared to AFDAS. The higher prevalence of large artery disease and small vessel disease in KAF suggested an alternative cause of ischemic stroke in a relevant proportion of patients with KAF despite anticoagulant treatment. These findings require further prospective testing.

## Supplementary Information

Below is the link to the electronic supplementary material.

Supplementary file1 (DOCX 14 KB)

## Data Availability

The data that support the findings of this study are available on request from the corresponding author. The data are not publicly available due to privacy or ethical restrictions.
