# Supplementary material for: Characteristics and outcomes of atrial fibrillation detected before and after acute ischemic stroke
Source: J Neurol. 2024 Aug 31;271(10):6856–65. doi: 10.1007/s00415-024-12671-z (PMC11447073; doi:10.1007/s00415-024-12671-z)
Supplement: Supplementary file 1 — Supplementary file1 (DOCX 14 KB) [file 415_2024_12671_MOESM1_ESM.docx]

**Supplemental table 1.**

| **Factor** | **SMD before PSM** | **SMD after PSM** |
| --- | --- | --- |
| age | -0.44 | 0.01 |
| gender | -0.03 | 0.02 |
| hypertension | -0.23 | 0.03 |
| smoking | 0.26 | 0.00 |
| CHA2DS2VASC | -0.43 | 0.02 |
| mRS at baseline | -0.75 | -0.01 |
| Diabetes | -0.04 | 0.03 |
| Dyslipidemia | -0.18 | -0.01 |
| Previous stroke | -0.44 | -0.03 |
| Previous ICH | -0.15 | 0.00 |
| NIHSS baseline | -0.45 | -0.02 |
| Heart failure | -0.50 | -0.03 |
